# Microsphere-mediated optical contrast tuning for designing imaging systems with adjustable resolution gain

**DOI:** 10.1038/s41598-018-33604-7

**Published:** 2018-10-12

**Authors:** Daniel Migliozzi, Martin A. M. Gijs, Gergely Huszka

**Affiliations:** Laboratory of Microsystems, École Polytechnique Fédérale de Lausanne, 1015 Lausanne, Switzerland

## Abstract

Upon illumination, a dielectric microsphere (μS) can generate a photonic nanojet (PNJ), which plays a role in the super-resolution imaging of a sample placed in the μS’s immediate proximity. Recent microscopy implementations pioneered this concept but, despite the experimental characterization and theoretical modeling of the PNJ, the key physical factors that enable optimization of such imaging systems are still debated. Here, we systematically analyzed the parameters that govern the resolution increase in the case of large-diameter (>20 µm) μS-assisted incoherent microscopy by studying both the illumination and the detection light paths. We determined the enhanced-resolution zone created by the μS, in which the detection system has a net resolution gain that we calculated theoretically and subsequently confirmed experimentally. Our results quantitatively describe the resolution enhancement mediated by the optical contrast between the μS and its surrounding medium, and provide concrete means for designing μS-enhanced imaging systems for several application requirements.

## Introduction

Photonic nanojets (PNJs) emerging from the shadow side of a dielectric micro-objects were studied intensively in recent years^1–6^, and have been employed in newly developed super-resolution microscopy techniques^7–9^. Because the PNJ was considered the main reason of the resolution increase in µS-assisted super-resolution microscopy, extensive characterization of this illumination mode was achieved by the microscopy community^10–20^. A variety of applications have been proposed for μS-assisted microscopy, from micro-topography measurement^21,22^ to live-cell imaging^23–25^, with implementations employing large μSs (>20 μm), because they provide an increased field-of-view and are easier to handle^26–29^. More recently, however, it has been proposed to revise and standardize the resolution claimed in μS-assisted microscopy and was demonstrated that the experimentally observed resolution gains could not be explained solely by the creation of the PNJ^30^. Indeed, while small sample-μS distances (<1 μm) are required for super-resolution imaging^31–33^, multiple numerical simulations showed that, in such configurations, the focus of the light is projected to several tens of micrometers away from the shadow-side of the microsphere^34–36^. Moreover, for such small sample-μS distance, objects of different nature may constrain the imaging conditions (*e.g*. use of a specific immersion medium for live-cell imaging). Previous studies on large μSs reported the assessment of the effective field-of-view and magnification created by the μS^37,38^, or the effect of embedding in an elastomer^39^. However, as those analyses mainly focused on the PNJ illumination, no exact prediction about the gain in spatial resolution was reported. Therefore, to establish the best imaging conditions for incoherent microscopy where large diameter (>20 µm) µSs are employed, we (i) explored both the illumination and the detection path through the μS to derive the theoretical resolution gain for different immersion configurations, (ii) validated the results experimentally, and (iii) provided parameters for imaging system optimization based on quantitative predictions.

## Results

### Analysis of the illumination path

**Figure 1:**
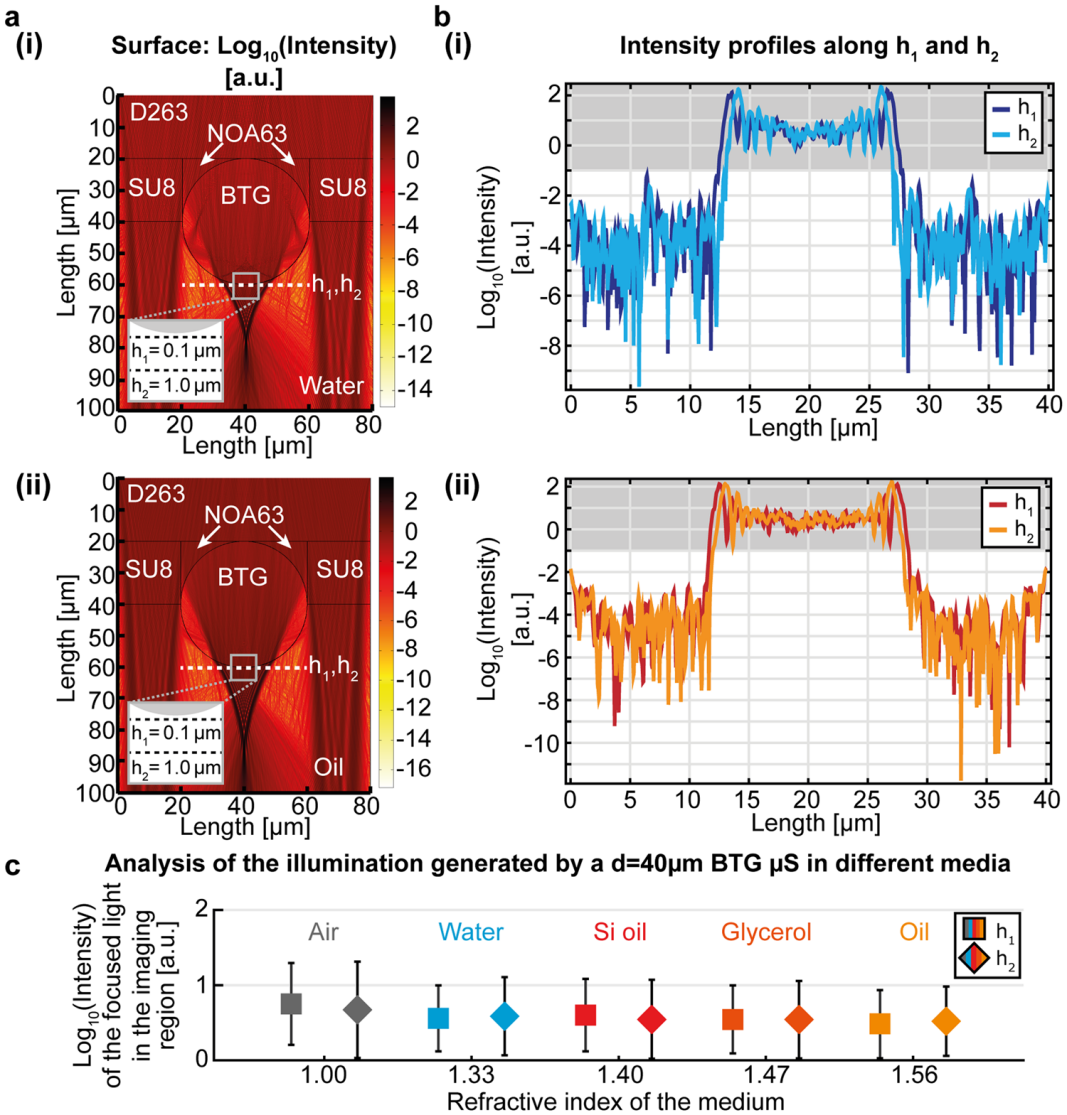
Analysis of the illumination profile in the imaging region of a μS. (**a**) FEM simulation of the PNJ generated by a BTG µS in (i) water- and (ii) oil-immersion upon flat-field illumination from the top. The surrounding optical environment (denoted by the material sections SU8, D263, and NOA63) of the µS is modelled based on the experimental configuration, as detailed in the Methods section. (**b**) Intensity profiles along the lines at h_1_ = 0.1 µm and h_2_ = 1.0 µm from the lower edge of the µS for (i) water- and (ii) oil-immersion. The grey-shaded regions indicate the focused light. (**c**) Comparison of the focused light generated by the PNJ along h_1_ and h_2_ in different immersion media. Data are plotted as median ± MAD of the intensity profile in the grey regions in (**b**) and Fig. S1, which correspond to the focused light in the imaging region.

To explore the contribution of the focused light in the illumination path, we studied its dependence on the optical contrast between the μS and the immersion medium. We used the finite element method (FEM) to calculate the electric field generated by a 40 μm diameter barium titanate glass (BTG) μS in either oil- or water-immersion medium upon illumination from the top (as detailed in the Methods section), and derived the light intensity values by multiplying the calculated electric field with its complex conjugate (Fig. 1a). Both systems project the focused light ~20 µm from the shadow-side of the µS but, since during imaging the sample must be in the vicinity of the µS, we investigated the intensity profiles within 1 μm from the μS, and found that in this position, they do not markedly differ in the two situations (Fig. 1b). Analysis of different μS materials and sizes in the most common immersion media (Figs 1, S1 and S2) shows that, as long as the optical contrast allows the formation of the PNJ, the intensity profiles close to the μS are very similar. This indicates that the PNJ illumination in the imaging region is very robust with respect to the optical contrast and will play a minor role in eventual resolution changes that would appear when varying this parameter.

### Analysis of the detection path

To analyze the detection path from the sample to the microscope objective, we used an analytical approach based on Fourier optics and ray tracing. Propagation of monochromatic light in homogenous media can be described by the Helmholtz equation^40^:

1 $$({\nabla }^{2}+{k}^{2})\psi ({\bf{r}})=0$$

where *ψ*(**r**) is the spatial part of the propagating field, $k=\frac{2\pi n}{\lambda }$ is its wave-number, with *λ* being its wavelength and *n* the refractive index of the medium. The general solution of Equation (1) in linear coordinates can be written as

2 $$\psi (x,y,z)={\int }_{-\infty }^{+\infty }{\int }_{-\infty }^{+\infty }{\hat{\psi }}_{0}({k}_{x},{k}_{y}){e}^{i({k}_{x}x+{k}_{y}y+{k}_{z}z)}d{k}_{x}d{k}_{y}$$

where *k*_*x*_, *k*_*y*_ and *k*_*z*_ are the component of the wave-vector $${\boldsymbol{(}}k={k}_{x}{\bf{x}}+{k}_{y}{\bf{y}}+{k}_{z}{\bf{z}})$$, and $${\hat{\psi }}_{0}({k}_{x},{k}_{y})={\int }_{-\infty }^{+\infty }{\int }_{-\infty }^{+\infty }$$$${\psi }_{0}(x,y){e}^{-i({k}_{x}x+{k}_{y}y)}dxdy$$ is the Fourier transform of the field at *z* = 0 (*i.e*. the object-plane). $${\hat{\psi }}_{0}({k}_{x},{k}_{y})$$ contains the spatial information about the object and is usually called angular spectrum^40^. With this approach, the spatial field solution can be interpreted as the sum of its plane wave components $${e}^{i({k}_{x}x+{k}_{y}y+{k}_{z}z)}$$, which are the eigenfunctions of the Helmholtz operator. Each of these plane waves carries some information about the spatial distribution of the object in the term $${\hat{\psi }}_{0}({k}_{x},{k}_{y})$$ and propagates at an angle *θ* with respect to the *z*-axis. This angle is given by the standard definition of the wave vector components in spherical coordinates:

3 $$\begin{array}{rcl}{k}_{x} & = & k\,\sin \,\theta \,\cos \,\phi \\ {k}_{y} & = & k\,\sin \,\theta \,\sin \,\phi \\ {k}_{z} & = & k\,\cos \,\theta \end{array}$$

where *θ* and *φ* are the polar and the azimuthal angle. The number of plane waves collected by the optical system determines the amount of spatial information retrieved about the original object, which explains why the maximum *θ*_obj_ that can be collected by an objective (*i.e*. half of the acceptance angle) limits the spatial resolution of the optical system in accordance with the standard definition of Abbe’s resolution:

4 $$R=\frac{\lambda }{2{{\rm{NA}}}_{{\rm{obj}}}}$$

where ${{\rm{NA}}}_{{\rm{obj}}}=n\,\sin \,{\theta }_{{\rm{obj}}}$ is the numerical aperture of the objective. Methods to artificially increase the angular spectrum collected by the detection system are used in techniques such as I5 M^29^ and SIM^30^.

**Figure 2:**
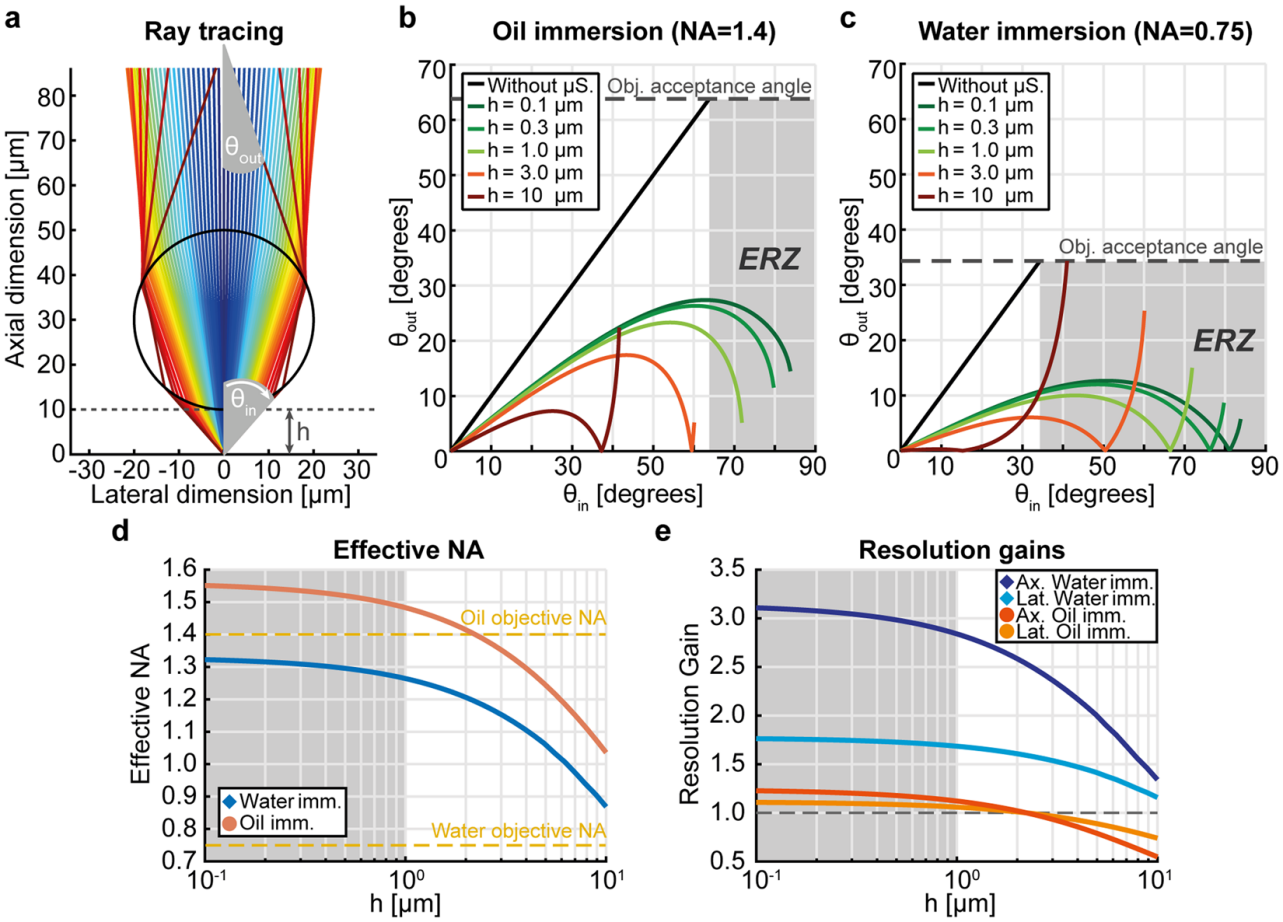
Enhanced-resolution zone (ERZ) created by the µS and estimation of the resolution gain. (**a**) Rays emerge from a point sample (located at the origin), and propagate through a µS (*n*_*s*_ = 1.95) placed in a homogenous medium (*n*_*m*_ = 1.56) up to the microscope objective (not shown). The sample-µS distance is marked with *h*; *θ*_*in*_ indicates the angle of a ray entering the µS, while *θ*_*out*_ indicates the angle of the corresponding outcoming ray. The color gradient indicates different propagating rays. (**b**,**c**) Relation between *θ*_*in*_ and *θ*_*out*_ for an oil- and a water-immersion objective, respectively, based on the model shown in (**a)**. Green curves indicate the sample-µS distances for which the illumination of the sample through the µS is the most suitable thanks to the photonic nanojet effect. The experimental angular acceptance of each objective is marked with a dashed line. The grey area shows the *ERZ*, where the µS mediates the collection of light coming from higher angles *θ*_*in*_ compared to the objective alone. (**d**) Effective NA in the presence of the µS for several sample-µS distances (*h*) as calculated from the configurations showed in Fig. 1. The dashed lines indicates the nominal NAs of the objectives without use of the µS. (**e**) Lateral and axial resolution gains. The dashed line indicates the performance of the objectives in the absence of the µS. The grey areas indicate the sample-µS distances for which the illumination of the sample through the µS is the most suitable thanks to the photonic nanojet effect.

Following this approach, we modelled the propagation of each individual wave originating from a point sample for a μS much larger than *λ* (Fig. 2a). Each wave propagates away from the sample at a certain angle with respect to the sample-μS axis, then it enters the μS by refraction, propagates inside the μS and exits again by refraction. In this procedure, the μS can be interpreted as a non-linear operator that transforms an input angle *θ*_in_ into an output angle *θ*_out_, both measured with respect to the optical axis. This operator depends on four parameters: the distance *h* between the sample and the μS; the diameter *d* of the μS; the refractive indices of the μS (*n*_s_) and of the surrounding medium (*n*_m_). By applying Snell’s law to the refraction points for each ray, and linear propagation otherwise, we calculated the functional relation between *θ*_in_ and *θ*_out_ for a BTG μS (*d* = 40 µm, *n*_s_ = 1.95) immersed in oil (*n*_m_ = 1.56) or in water (*n*_m_ = 1.33), for sample-μS distances ranging from 0.1 to 10 µm (Fig. 2b,c). The method for this calculation is detailed in the Methods section. The relation between *θ*_in_ and *θ*_out_ is very different for oil and water immersion. However, both situations lead to a decrease of *θ*_out_ compared to the absence of the μS. Parallel, the objective acceptance angles for an oil-immersion objective with NA = 1.4 and a water-immersion objective with NA = 0.75 are shown. The larger angles that are guided back to the objective by the μS generate an enhanced-resolution zone (ERZ) in which the amount of collected angular spectrum is increased. This translates into a net increase of the NA of the system (Fig. 2d), which results in a net gain for the lateral resolution as derived from equation (4):

5 $$G=\frac{{R}_{0}}{{R}_{\mu S}}=\frac{{n}_{m}\,\sin \,{\theta }_{\mu S}}{N{A}_{obj}}$$

where *θ*_μS_ is the maximum *θ*_in_ refracted by the μS that stays within the acceptance cone of the objective. For the axial resolution, which scales as NA^−2^, the gain is even higher and corresponds to *G*^2^. In Fig. 2e we show both lateral and axial resolution gains for the oil- and water-immersion objectives considered in Fig. 2b,c. We find that the resolution gain is markedly different when using a μS in either water or oil medium, and that much more gain is expected theoretically when using the μS in water. Moreover, as the gain is uniform in this region, no very precise location of the sample is needed to get the same resolution enhancement. This guarantees the imaging robustness which is very useful in optical system design.

**Figure 3:**
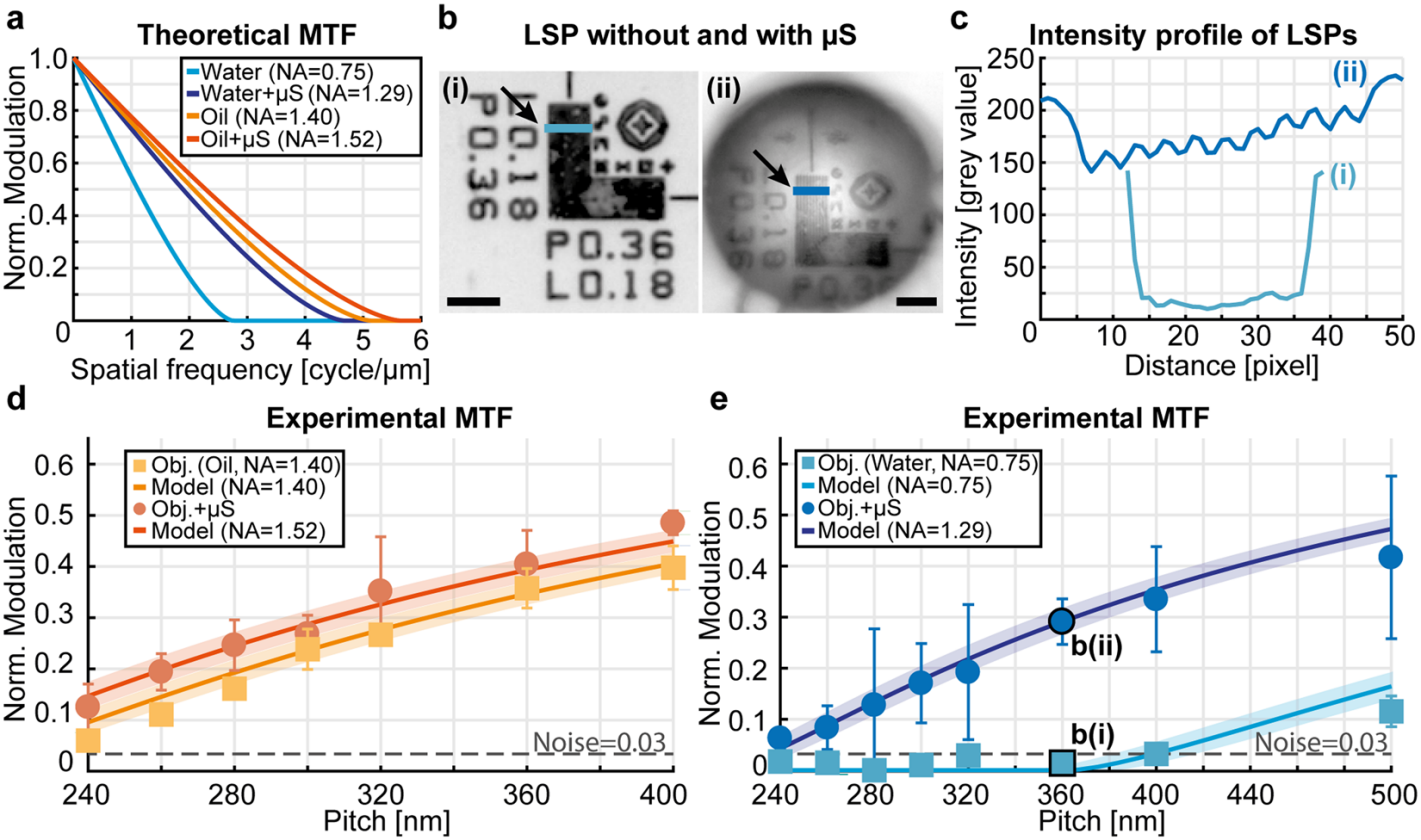
Experimental confirmation of the predictions for different configurations. (**a**) Modulation transfer function (MTF) calculated at λ = 545 nm, for water and oil immersion objectives without and with use of a µS. The NA in the presence of the µS is derived from Fig. 2a, for a sample-µS distance lower than 1 µm. (**b**) Examples of the imaged line-space micro-patterns (LSMPs) without (i) and with (ii) the µS, respectively. Scale bars: 5 µm. (**c**) Intensity profiles along the lines marked by the arrows in (**b**). (**d**,**e**) Experimental MTF for oil- and water-immersion, respectively, measured on Si-based LSMPs of several pitches. Data are plotted as median ± MAD of the modulation peaks for each individual line of a given LSMP (*n* = 20 per LSMP). The solid lines with the shaded bands show the theoretical MTF for λ = 545 ± 20 nm, which corresponds to the band-pass filter of the microscope system. The dashed line marks the measured noise level of the imaging system.

To verify the theoretical predictions, we compared the measured and calculated spatial frequency coverage by imaging line-space micro-patterns (LSMPs), which was shown as a suitable characterization method of optical systems for both coherent^41^ and incoherent^42^ imaging. The spatial frequencies present in the object are partially filtered by the optical system, so that the contrast between the features in the object is attenuated in the image. In incoherent imaging, the intensity modulation in the object is transferred to an intensity modulation in the image by the modulation transfer function (MTF) of the optical system, which is given by the modulus of the Fourier transform of the point spread function (PSF)^40^. In the case of microscope objectives, for which the PSF is the well-known Airy disk, the MTF is

6 $${\rm{MTF}}=\frac{2}{\pi }(f-\,\cos \,f\,\sin \,f)$$

where $f=\arccos (\frac{\lambda }{2{\rm{NA}}}u)$ is the normalized spatial frequency, *u* being the linear spatial frequency. In Fig. 3a, we calculated the MTF for the two objectives considered in Fig. 2, in the presence and absence of a μS. The range of *λ* considered for this calculation was 545 ± 20 nm, corresponding to the experimental band-pass filter placed behind the light source. Similarly, the NA considered in the presence of the μS was the one obtained for short sample-μS distance (*i.e*. for the sample laying within the imaging region). The maximum spatial frequency that is predicted to be resolved by the objective (*i.e*. MTF(*u*) > 0) is increased in the presence of the μS for both oil- and water-immersion. However, the water-immersion objective is expected to gain more than the oil-immersion objective. To illustrate the modulation enhancement below the diffraction limit of the optical system (λ/2NA = 545 nm/2*0.75 = 363 nm), we show an LSMP of 360 nm-pitch in the absence and presence of the μS in Fig. 3b and their intensity profiles in Fig. 3c, which shows that the LSMP peaks becomes resolvable when imaged through the water-immersed μS. The predictions were confirmed by the measurements on several LSMPs (Fig. 3d,e), where the expected behavior of the objective alone was confirmed and the predicted spatial frequency coverage in the presence of the μS followed the predictions perfectly. The Abbe resolution limit corresponds to the point at which the MTF crosses the horizontal axis, and we also report the noise level of the optical system (dotted lines in Fig. 3d,e) as a reference, which further confirms that the null modulation predicted below the diffraction limit without the μS (light-blue line) lies indeed within the noise region (light-blue squares) as expected. We report the image of a single line in Fig. S3 to elucidate the difference between the resolution of the optical system and its minimal detectable feature.

## Discussion

**Figure 4:**
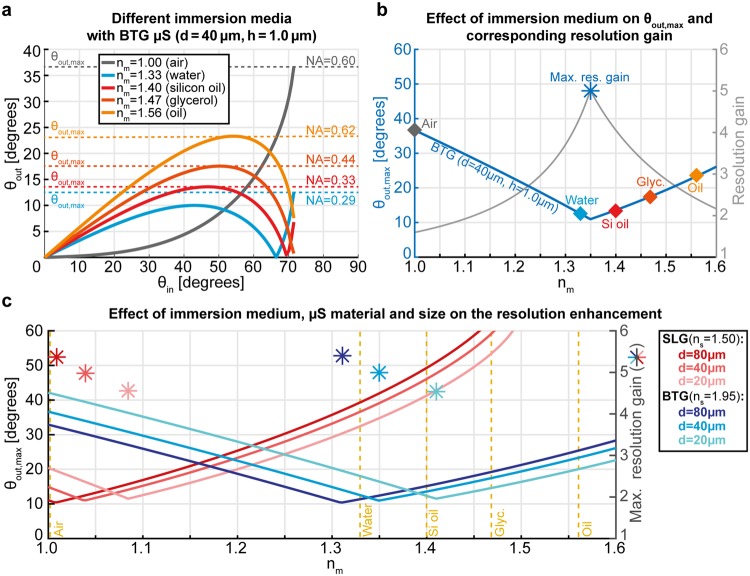
Effect of the optical contrast and the µS size on the resolution gain. a, Relation between *θ*_*in*_ and *θ*_*out*_ for the most common immersion media. Dashed lines indicate the maximum output angle (*θ*_*out,max*_) and the corresponding minimum NA needed for the objective to collect the rays from all the input angles. (**b**) *θ*_*out,max*_ as a function of the refractive index of the medium (*n*_*m*_) for the same µS as in (**a)**. The values corresponding to the five media from **a** are marked with diamonds. The resolution gain for a given *n*_*m*_ is shown on the secondary Y-axis. The maximum value of the gain corresponds to the location of the minimum *θ*_*out,max*_, and is marked with an asterisk. (**c**) Solid lines represents the *θ*_*out,max*_ for soda-lime glass (SLG) and barium titanate glass (BTG) µSs, for several μS diameters. Asterisks (*) indicate the maximum resolution gain for the given combination of medium, µS material and diameter. Vertical dashed lines indicate the refractive indices of the most common immersion media.

This systematic characterization of the resolution gain mediated by the μS enables to explore the properties of the resulting imaging system in a detailed manner, and optimization of the imaging conditions can then be assessed with the described model. For instance, it can be used to predict the resolution gain for configurations with various immersion media, μS materials and sizes. We demonstrate the potential of the quantitative predictions of the model in Fig. 4. We calculated the relation between the input and the output angle for the most common immersion media (Fig. 4a). In order not to lose generality, we did not consider the optical glue and the supporting glass for these calculations, because they can be different in other implementations of the technique^12,15,26,33–35^. On the same graph, we report the output ${{\rm{NA}}}_{{\rm{out}}}={n}_{{\rm{m}}}{\theta }_{\mathrm{out},\max }$ for each condition, where *θ*_*out,max*_ is the maximum output angle (*i.e*. the minimum acceptance angle needed for the objective to collect the entire spatial information). The lower NA_out_, the larger the resolution gain that can be obtained. From air (lowest refractive index) to oil (highest refractive index) the behavior changes in a non-monotonic way, since increasing *n*_m_ does not always result in lowering NA_out_. This demonstrates that the optimal resolution gain has a non-trivial dependence on the contrast between the refractive indices of the medium and the μS. To study this effect, we calculated *θ*_*out,max*_ as a function of *n*_m_ in the range 1–1.6 (Fig. 4b). A minimum is clearly visible for *θ*_*out,max*_, at which the resolution gain reaches a maximum. Finally, we studied this behavior for two common glass types for a large range of μS (20–200 μm) and showed a representative subset of *θ*_*out,max*_ (lines) and resolution gains (stars) in Fig. 4c. We observe that the position of the maximum resolution gain shifts mainly when changing the μS material (>10%), and only slightly when varying the μS size in the μm range (<10%). This calculations of the resolution enhancement provide a quantitative design tool for optimization of μS-assisted imaging systems: for instance, when the sample requires no immersion, soda-lime glass (SLG) μSs give a higher gain; conversely, when studying living cells in water-based media, BTG μSs are more suitable.

In conclusion, these results quantitatively describe the resolution gain mediated by the μS as a function of the physical parameters of the optical system, and provide predictions for several configurations of μS materials and immersion media, offering a method to optimize microscopy configuration for different application requirements based on μS-assisted imaging.

## Methods

### Calculations for comparing the photonic nanojet in water- and oil-immersion systems

To analyze the PNJs created by the water- and the oil-immersion system, finite element method (FEM) simulations were carried out in COMSOL Multiphysics software. Propagation of 545 nm light (corresponding to the illumination setup) was modelled based on the configuration of the chip used for the experiments. The chip consisted of a D263 glass substrate (*n*_*D263*_ = 1.525) on which a BTG µS (*n*_*BTG*_ = 1.950) was placed between the SU8 (*n*_*SU8*_ = 1.575) sidewalls. The cavities between the µS and the D263 were filled with NOA63 optical glue (*n*_*NOA63*_ = 1.560). The modelling of the surrounding of the microsphere was necessary in order to establish the same illumination conditions as in our experimental setup. However, the same results can be obtained if the SU8 and the D263 glass are not considered, as the former is not involved in the investigated light path, while border of the latter is reached by the light perpendicularly (*i.e*. Snell’s law ensures that in this case the light can travel forward without refraction). On the other hand the optical glue may play a minor role, as its refractive index deviates from the one of the D263 glass’ by less than 0.04. In order to obtain a precise solution, we did not neglect this difference. The immersion medium was a changing parameter (*n*_*air*_ = 1.000, *n*_*water*_ = 1.333, *n*_*SiOil*_ = 1.400, *n*_*glycerol*_ = 1.470, and *n*_*oil*_ = 1.560). The following scalar equation was used to study transverse electric waves in this two dimensional model:

$$\nabla \times (\nabla \times E)-{k}_{0}^{2}{\varepsilon }_{r}E=0$$

where *k*_0_ is the free-space wave number, *ε*_*r*_ = (*n*-i*k*)^2^ is the relative permittivity, expressed with the refractive index *n* and its imaginary part *k*. In this model, the scattering boundary condition was used at all exterior boundaries, and the continuity boundary condition was used at all material interfaces. During meshing, the minimum element size was 10 nm, while the maximum element size of λ/4 was set to obtain a precise solution. After the model was solved, the normalized electric field was multiplied by its conjugate and the logarithmic of the intensity values were plotted (Figs. 1, S1 and S2).

### Analytical calculation of the light path through the microsphere

**Figure 5:**
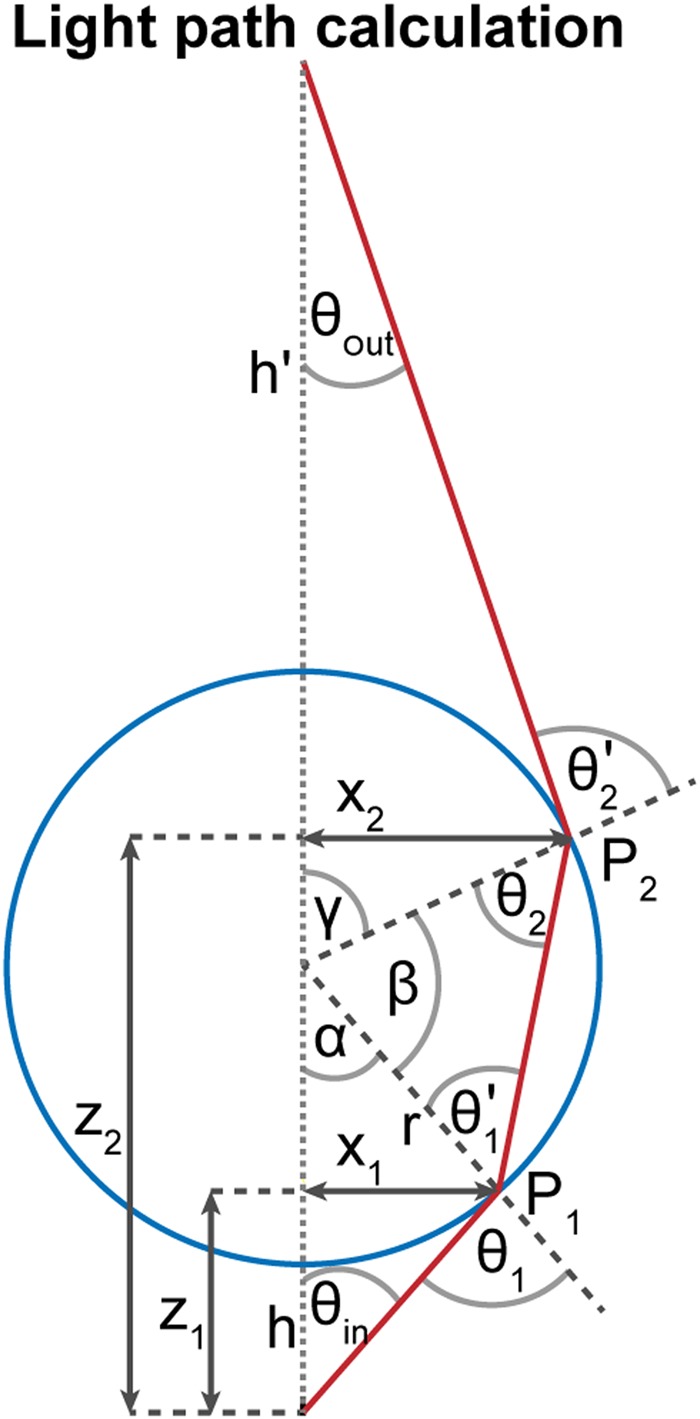
Notation for light path calculation. The blue circle and the red line mark the µS and the ray path, respectively. The dotted vertical line shows the sample-µS axis (*i.e*. the optical axis). The geometrical parameters are described in the text.

To quantitatively calculate the effect of the μS on the light cone rising from the sample, we used the property of cylindrical symmetry to reduce the calculation to a 1D problem (Fig. 5). Each ray rising from the sample can be fully described by its starting polar angle *θ*_in_ with respect to the sample-μS axis. With the notation used in Fig. 5, Snell’s law at the refraction points P1 and P2 imply

7 $$\begin{array}{cc}{n}_{{\rm{m}}}\,\sin \,{\theta }_{1}={n}_{{\rm{S}}}\,\sin \,{\theta }_{1}^{^{\prime} } & {n}_{{\rm{S}}}\,\sin \,{\theta }_{2}={n}_{{\rm{m}}}\,\sin \,{\theta }_{2}^{^{\prime} }\end{array}$$

Moreover, trigonometric properties imply

8 $$\begin{array}{cccc}{\theta }_{1}=\alpha +{\theta }_{{\rm{in}}} & {{\theta }_{2}}^{^{\prime} }=\gamma +{\theta }_{{\rm{out}}} & {\theta }_{1}^{^{\prime} }+{\theta }_{2}+\beta =\pi  & \alpha +\beta +\gamma =\pi \end{array}$$

Finally, simple geometry gives

9 $$\begin{array}{cccc}{x}_{1}=r\,\sin \,\alpha  & {z}_{1}=h+r(1-\,\cos \,\alpha ) & {x}_{2}=r\,\sin \,\gamma  & {z}_{2}=h+r(1-\,\cos \,\gamma )\end{array}$$

Equations (7–9) were used to calculate *θ*_out_ in the range of ${\theta }_{in}\in [0;\,\arcsin \frac{r}{r+h}]$, which corresponds to the angles that intercept the μS. In cases where the optical glue and the glass coverslip were taken into account, Snell’s law and simple geometry gives

$${n}_{glue}\,\sin \,{\theta }_{out}={n}_{m}\,\sin \,{{\theta }_{out}}^{^{\prime} }$$

Where *θ*_*out*_′ is the final output angle.

### Image acquisition

To perform the experimental control for the theoretical MTF, a custom imaging setup was built. First, a chip with fixed BTG microspheres was created. A D 263 M borosilicate glass (Menzel-Gläser, Germany) substrate of 22 × 22 × 0.15 mm^3^ was cleaned with oxygen plasma which was followed by 20 µm 3025 type SU8 (MicroChem, USA) coating. Then, 40 µm diameter wells were patterned into the SU8 layer with photolithography. After development, a 4 µl droplet of Norland Optical Adhesive 63 (NOA63, Norland Products, USA) was spread on the top of the chip. This was followed by a 20 minutes vacuum treatment to remove the air bubbles stuck in the wells of the SU8 layer. Subsequently, 38–45 µm diameter BTG (Cospheric, USA) microspheres were placed on the NOA63 layer. The microspheres were swiped over the surface multiple times until they got located in the wells. The excess amount of microspheres was removed to prevent them acting as a spacer during imaging. Finally, the chip was exposed to UV light until an accumulated dose of 4.5 Joules/cm^2^ was reached, which is required for curing the NOA63 optical glue. For the experiments, this chip was placed upside down onto the sample, *i.e*. the BTG microspheres came into close contact with the sample. The imaged sample was a silicon-based microscope calibration target (MetroBoost, USA), which contained line-space micro-patterns (SiO_2_ – PolySi, respectively) with various pitch between 240–500 nm. The sample with the chip on top was imaged with an Axio Imager M2m upright optical microscope, equipped with HAL100 halogen light source (Zeiss, Germany). A filter cube with a band-pass (524–565 nm) excitation filter and an 80T-20R beam splitter (both from AHF, Germany) was placed in the optical path. For the oil-immersion experiments, a 63x, Na = 1.4 objective (Zeiss, Germany) was used. The images were captured with an AxioCam MRm (Zeiss, Germany) that had 6.45 µm × 6.45 µm pixel size. This resulted in mapping 102 nm of the sample into 1 pixel. For the water-immersion experiments, a 40×, NA = 0.75 objective (Zeiss, Germany) was used in combination with a DMK31BF03.H (TIS, Germany) camera that had 4.65 µm × 4.65 µm pixel size, which resulted in mapping 116 nm to 1 pixel. Note that, when present, the BTG microsphere introduces an extra ~2× magnification to the system.

### Quantification of the experimental MTF

After acquisition, the images were stored as 8 bit grayscale pictures. To determine the modulation of the recorded signal, the average intensity values along a 5 pixel-wide line (crossing the line-space micro-patterns) were extracted (Fig. 4b,c). The structure of 8 bit grayscale picture is constructed in a way that the value 255 belongs to a fully white pixel, meanwhile 0 marks a totally black one. Because of the material properties of the imaged sample, the background looked bright on the images and the lines were seen as dark lines. Corresponding to that, when plotting the intensity profile, the signal looked like a step from higher value to lower ones, then the modulation could have been seen and then a step back to the starting level (Fig. 4c). The modulated region was analysed by detecting peaks and valleys and calculating all amplitudes (*a*) from these values. Because of the structure of the sample, this meant 9–11 values (one per line), from which we calculated *a*′ = median(*a*) and *a*′_*error*_ = MAD(*a*), where *a* is the vector of the amplitude values. Then the step (*s*) was measured. Subsequently, the modulation (*M*) was calculated as:

$$M=\frac{{I}_{{\rm{\max }}}-{I}_{{\rm{\min }}}}{{I}_{{\rm{\max }}}+{I}_{{\rm{\min }}}}=\frac{a^{\prime} }{2s-a^{\prime} }$$

To guarantee the same normalization of the modulation values as the theoretical MTF, which is always normalized at unity at zero spatial frequency, another area of the sample was imaged, where the same two materials had a common border but without any modulation pattern (*i.e*. with a null spatial frequency = 0). This showed how the imaging system could map the maximum observable modulation (*M*_0_). From this measurement, *M*_0_ was calculated as:

$${M}_{0}=\frac{step-baseline}{step+baseline}$$

where *step* was the average grey value over 10 pixel in the SiO_2_ (to measure maximum dark value) and *baseline* was the same in PolySi (to measure maximum bright value). The final normalized modulation (*M′*) that is shown in Fig. 4d,e was derived as *M′* = *M*/*M*_0_.

The noise level modulation was calculated by applying the same methodology in 24 uniform regions (*i.e*. without the presence of any microstructured edge) for each immersion type, then averaged and normalized.

## Electronic supplementary material

Supplementary material
