# Supplementary material for: Microsphere-mediated optical contrast tuning for designing imaging systems with adjustable resolution gain
Source: Sci Rep. 2018 Oct 12;8:15211. doi: 10.1038/s41598-018-33604-7 (PMC6185990; doi:10.1038/s41598-018-33604-7)
Supplement: Supplementary file 1 — Supplementary material [file 41598_2018_33604_MOESM1_ESM.docx]

**Microsphere-mediated optical contrast tuning for designing imaging systems with adjustable resolution gain**

Daniel Migliozzi, Martin A. M. Gijs*, and Gergely Huszka

Laboratory of Microsystems, École Polytechnique Fédérale de Lausanne, 1015 Lausanne, Switzerland

*Corresponding author: martin.gijs@epfl.ch

**Supplementary Information**

**Finite element method (FEM) simulations of the photonic nanojets**

In this supplementary material, we show FEM simulations that support our findings reported in the main manuscript. The description of the preparation process of these results can be found in the methods section. In Fig. S1, which supports Fig. 1 in the main manuscript, the BTG µS is placed in different immersion media (left column in the figure). These media are the most commonly used in optical microscopy techniques. The surroundings of the µS is modelled based on the chip that was used for the experimental verification. We analyzed the illumination generated by the µS at two different distances (h1=0.1 µm and h2=1.0 µm) from the shadow side of the µS (right column in the figure). These positions correspond to the location of the sample during the imaging. Analysis of the intensity profiles show that there is no significant difference in the focused light region (shaded area on the plots). Numerical results of this analysis are shown in Fig 1.c.

To extend our study on the generated illumination, we investigated how the material and the size of the µS influences the intensity of the focused light. Simulations with soda-lime glass (SLG) and barium titanate glass (BTG) were carried out for both 20 µm and 40 µm diameter µSs. The intensity profiles were generated as described earlier. Measurements were made at the same distances as previously and the numerical results are showed in Fig. S2. In all cases, the calculated values for h1 and h2 are almost identical, which indicates that the illumination is robust in the imaging region, i.e. closer than 1 µm from the µS. Furthermore, the focused light intensity does not change significantly for BTG µSs, which demonstrates that there is no size dependence in this case. As for SLG, a decreasing tendency is observable. This can be explained by the insufficient optical contrast, since nSLG=1.50: for low refractive index-media, such as air and water, the optical contrast is still sufficient to generate the PNJ, and the intensity values are almost identical in the imaging region. However, as the contrast decreases, the focusing effect fades away. When the medium has almost the same refractive index (e.g. glycerol) as the µS, there is no focusing effect at all; therefore, the light intensity is not increased. If the refractive index of the media is further increased (e.g. oil), even deflection of the light can occur, which decreases the original intensity. This behavior was observed independently from the size of the SLG µS. We conclude that if the optical contrast is high enough to create a PNJ, then the illumination in the imaging region is independent from the immersion medium.


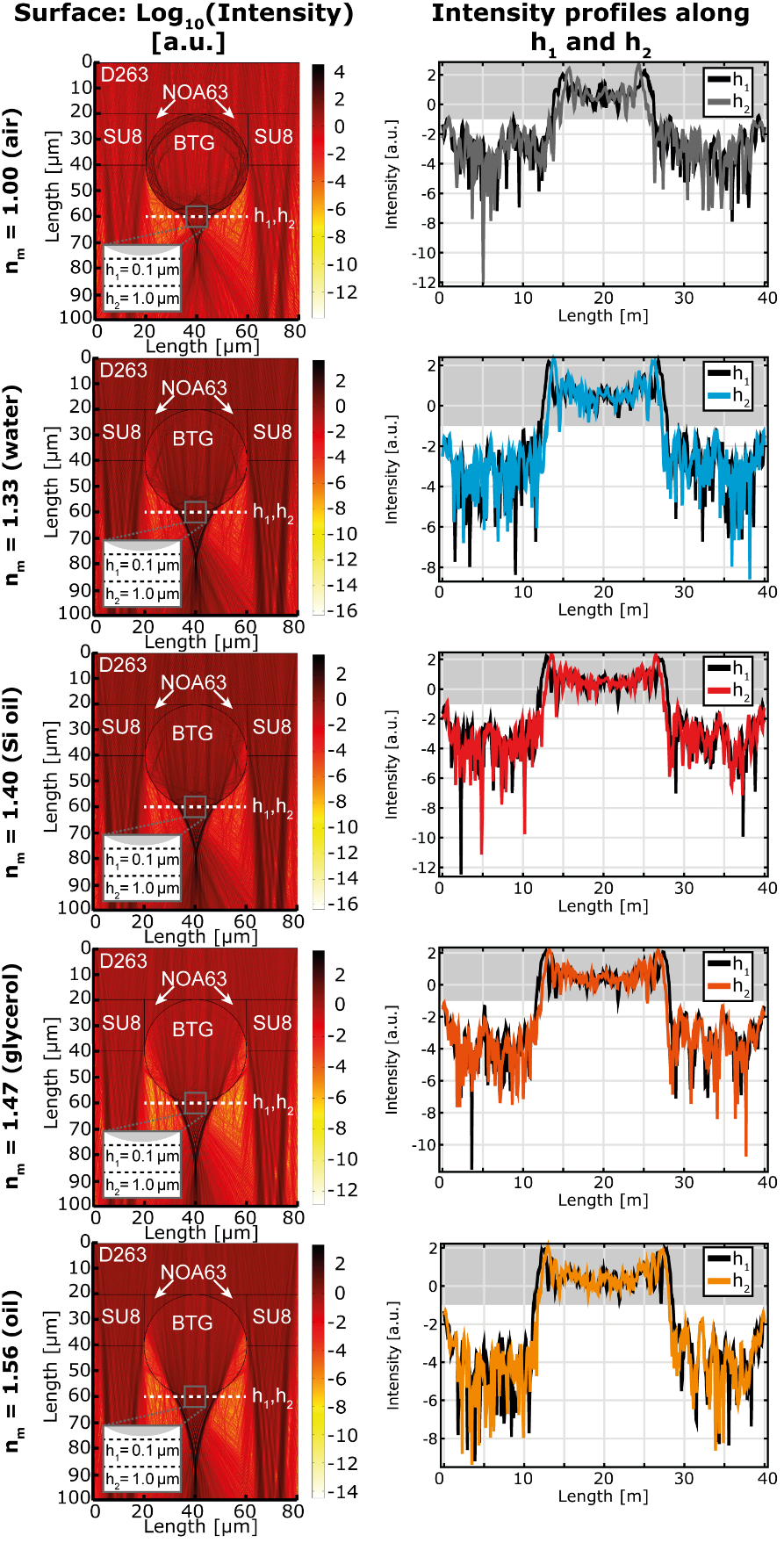


**Fig S1 | Analysis of the illumination profile in the imaging region.** FEM simulation of the PNJs generated by using different immersion media (left column) upon flat-field illumination from the top. Two measurement lines, h_1_ and h_2_ are located at 0.1 µm and 1.0 µm distance from the lower edge of the µS, respectively. The corresponding intensity values along the two lines are shown on the plots (right column). Note, that the vertical axis has logarithmic scale.


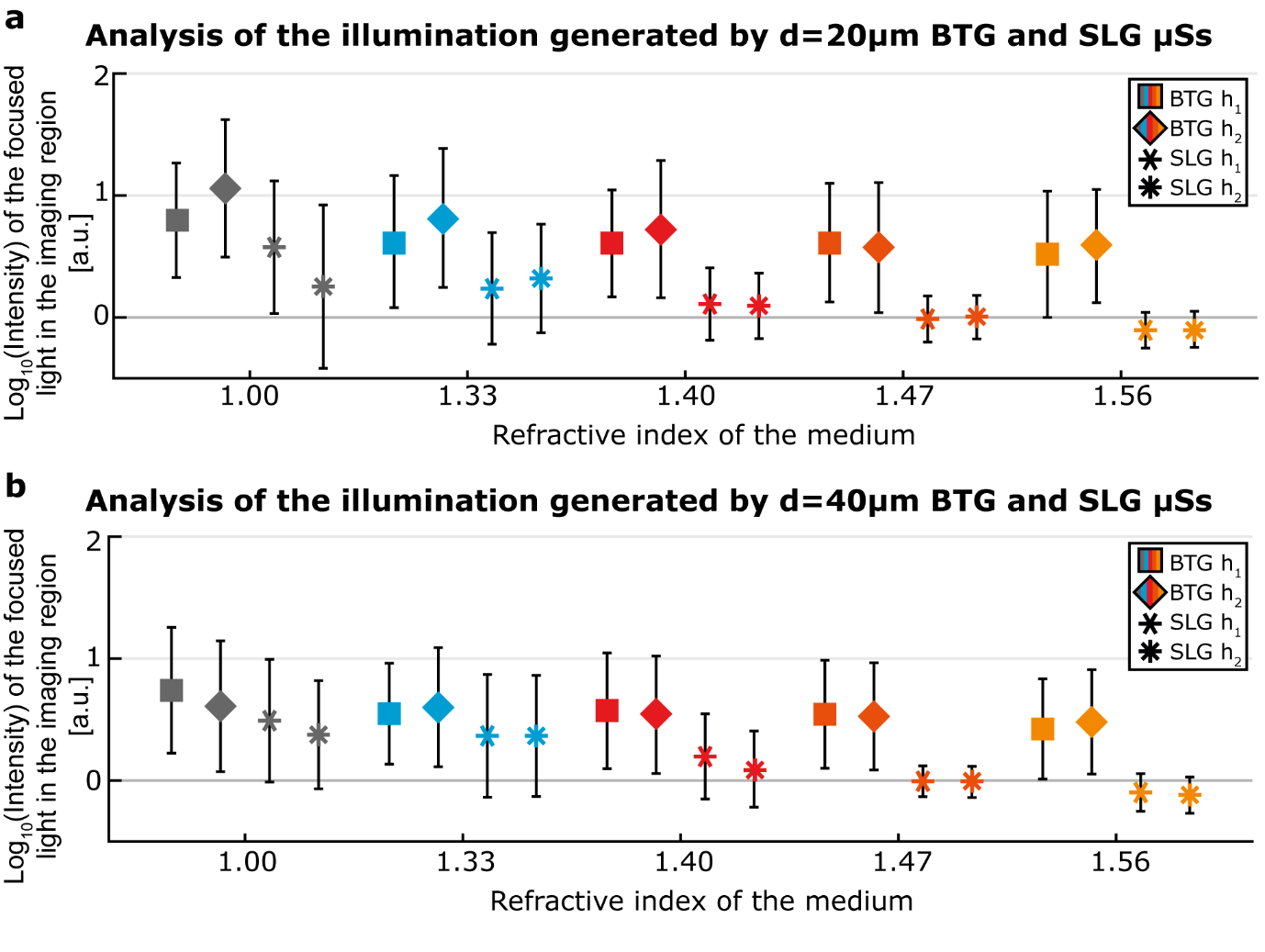


**Fig S2 | Analysis of the illumination for BTG and SLG µSs with various diameters. a, b** Focused light intensity values for BTG and SLG µSs with diameters d=20 µm and d=40 µm are calculated with the same method as shown in Figure 1 and S1. Data are plotted as median ± MAD of the intensity profile of the focused light in the imaging region.

**Single-object imaging *vs* resolution**


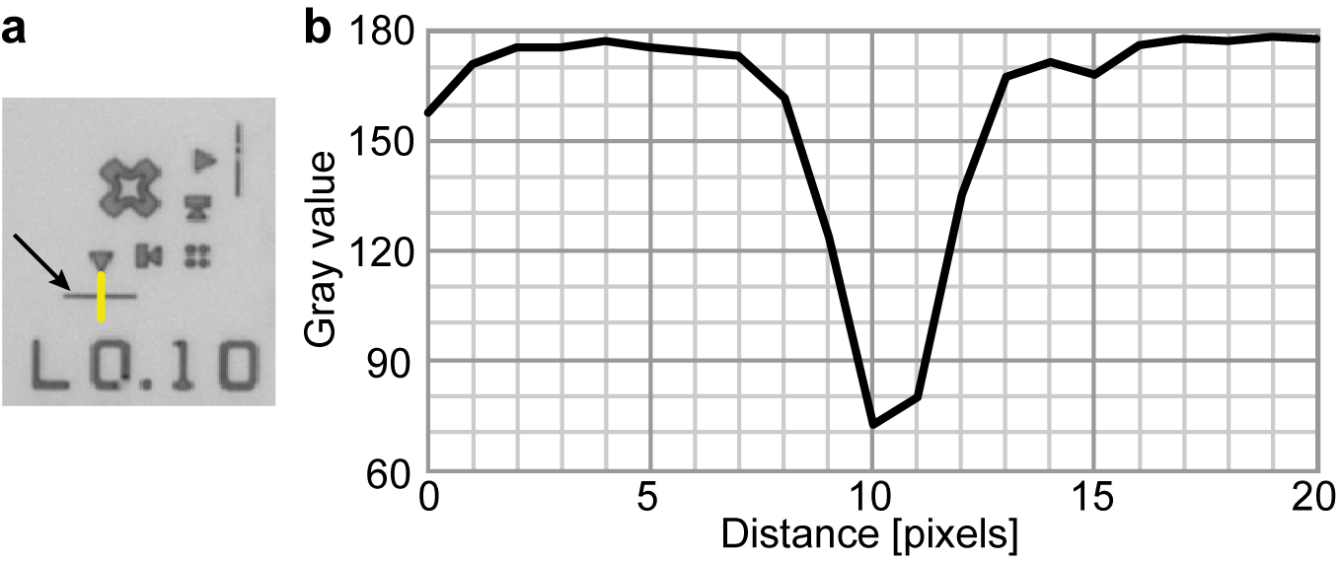


**Fig S3 | Single-line imaging.** **a**, Silicon-based microscope calibration target (described in the methods) imaged through a 63×, NA=1.4, oil-immersion objective. Arrow marks a single 100 nm-wide line. **b**, Intensity profile along the yellow line marked in **a**.

To clarify the difference between the resolution of the optical system and the size of the minimal detectable feature, in Fig. S3 we show a single 100 nm-wide line imaged with a 63×, NA=1.4, oil-immersion objective. The Abbe resolution of this optical system is 545/2*1.4=195 nm, which is almost double the size of the structure detected. In fact, objects smaller than the resolution limit can easily be detected with standard optical systems when completely isolated from other objects. For this reason, the MTF method uses small objects close to each other (*e.g.* regularly-spaced lines) to establish the resolution of an optical system.
